# Evaluation of Sampson equation for LDL-C in acute coronary syndrome patients: a Chinese population-based cohort study

**DOI:** 10.1186/s12944-022-01648-4

**Published:** 2022-04-19

**Authors:** Jiayu Li, Yanguo Xin, Jingye Li, Meng Meng, Li Zhou, Hui Qiu, Hui Chen, Hongwei Li

**Affiliations:** 1 Department of Cardiology, Beijing Friendship Hospital, Capital Medical University, No.95 Yong’an Road, Xicheng District, 100050 Beijing, China; 2 Department of Internal Medical, Medical Health Center, Beijing Friendship Hospital, Capital Medical University, Beijing, China; 3 Beijing Key Laboratory of Metabolic Disorder Related Cardiovascular Disease, Beijing, China

**Keywords:** LDL-C, Hypercholesterolemia, Sampson equation, Chinese

## Abstract

**Objective:**

Low-density lipoprotein cholesterol (LDL-C) is an important cardiovascular disease marker that is used to estimate the risk of acute coronary syndrome in patients. The Sampson equation is an accurate LDL-C equation, but its application in Chinese patients is unclear.

**Methods:**

This study enrolled 12,989 consecutive Chinese patients with the acute coronary syndrome (ACS), LDL-C levels were determined by direct standard method and two indirect equations (Friedewald and Sampson). The detection accuracy and consistency of these two equations were compared in patients classified by triglyceride (TG). In addition, the efficiency of the Sampson equation was also evaluated in patients with different comorbidities.

**Results:**

Patients were divided into six groups according to TG level, and indicated that the Sampson formula was more accurate than the Friedewald formula in all TG spectrums (*P* < 0.001). The Friedewald formula may underestimate the risk in patients with TG > 400 mg/dL, especially in TG > 800 mg/dL group (r: 0.931 vs. 0.948, 0.666 vs. 0.898, respectively). Compared with the Friedewald equation, the Sampson equation showed more advantages in female, age ≥ 65, body index mass (BMI) < 25, non-smoker, and non-diabetes (0.954 vs. 0.937, 0.956 vs. 0.934, 0.951 vs. 0.939, 0.951 vs. 0.936, and 0.947 vs. 0.938, respectively) than those in male, age < 65, BMI ≥ 25, smoker, and diabetes.

**Conclusions:**

Compared with the Friedewald equation, the Sampson equation is more accurate for LDL-C evaluation in Chinese patients diagnosed with ACS, especially in patients with hypertriglyceridemia even in those with TG > 800 mg/dL. Additionally, the Sampson equation demonstrates greater accuracy even in subgroups of various baseline characteristics and comorbidities.

## Introduction

Hypercholesterolemia is the initiator of vessel damage and ultimately leads to atherosclerosis and coronary heart disease (CHD) [1]. Elevated LDL-C levels usually come with higher CHD risk [2–4]. A study including more than 90,000 individuals indicated that every 1mmol/L reduction in LDL-C could contribute to a 21% reduction in major vascular events [5]. In addition, plenty of evidence reports that the levels of LDL-C contribute to the prediction of prognosis in CHD patients. Various clinical agents targeting LDL-C, such as statins and proprotein convertase subtilisin/kexin type 9 (PCSK9) inhibitors have been recommended by guidelines [6]. For secondary prevention in very-high-risk patients, an LDL-C reduction of ≥ 50% from baseline and an LDL-C goal of < 1.4mmol/L are recommended. Thus, an accurate estimation of LDL-C is necessary [6].

Various methods have been applied, either calculation methods or laboratory testing methods, for the determination of LDL-C, including the Friedewald equation, Martin equation, β-quantification, homogeneous assay, electrophoresis, and sequential and density-gradient ultracentrifugation [7–13]. Currently, the Friedewald equation is the most widely used indirect technique in the clinical laboratory: LDL-C = total cholesterol-HDL-C-TGs/5. The main source of bias in the Friedewald equation comes from TGs/5, therefore, the accuracy will decrease when plasma triglycerides > 4.52 mmol/L or when collecting specimens in a non-fasting state [14, 15]. To overcome the disadvantages of this equation, Martin et al. [10] developed a new method for estimating LDL-C using an adjustable factor for triglycerides, and some clinical evidence indicated that the Martin method showed a better concordance than the Friedewald Eq. 1[3]. Unfortunately, the advantage of the Martin equation does not exist for those with triglycerides > 400 mg/dL. The β-quantification procedure for LDL-C measurement was considered as the standard method by the National Cholesterol Education Program (NCEP) Lipoprotein Measurement Working Group [16]. However, specialized equipment and large serum volume are required for this method, additionally, the procedure is time-consuming; Homogeneous assays, a method for measuring serum LDL-C adopting enzymatic selective protection, are the most widely used laboratory testing method of LDL-C in Chinese hospitals at present, exerting huge economic pressure on clinical laboratories.

In 2020, Maureen and colleagues developed a new equation, the Sampson equation, for the calculation of LDL-C in patients with or without hypertriglyceridemia (≤ 800 mg/dL)[17]. However, the efficiency of the Sampson equation has not been verified in Chinese patients. In this clinical study, the Sampson equation was compared with the traditional Friedewald equation and homogeneous assay for estimating LDL-C in acute coronary syndrome.

## Materials and methods

### Methods

**Fig. 1:**
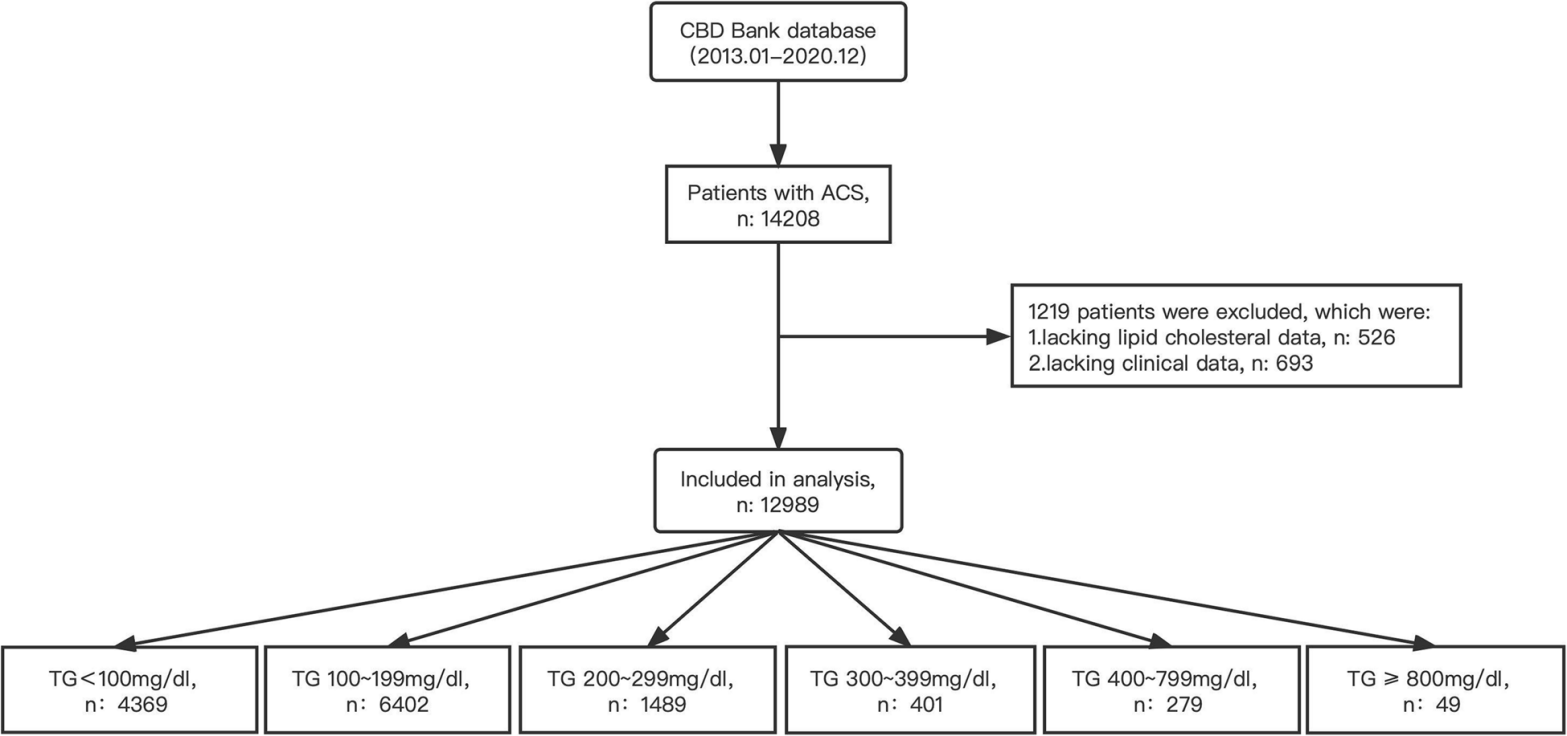
Flow chart of study subject enrollment. (CBD, Cardiovascular Center of Beijing Friendship Hospital Database; ACS, acute coronary syndrome; TG, triglyceride)

In this retrospective study, the lipid profiles were evaluated in 12,989 consecutive patients (consists of 4559 females and 8430 males, aged 25–99 years) with ACS in China, from 1 to 2013 to 1 December 2020, using a database from the Cardiovascular Center of Beijing Friendship Hospital Database Bank (CBD Bank). As illustrated in Fig. 1, the medical records of 14,208 ACS patients were screened, and then 1219 patients were excluded according to the following exclusion criteria: (1) lacking lipid cholesterol or triglyceride data; (2) lacking clinical data, (3) pregnant patients, (4) patients suffering infectious diseases or patients receiving cancer therapies that could affect blood lipid. According to the triglycerides level, 12,989 patients were stratified into six groups: the TG < 100 mg/dL group (*n* = 4369), the TG 100–199 mg/dL group (*n* = 6402), the TG 200–299 mg/dL group (*n* = 1489), the TG 300–399 mg/dL group (*n* = 401), the TG 400–799 mg/dL group (*n* = 279), the TG ≥ 800 mg/dL group (*n* = 49) (Fig. 1).

### Lipid measurements

The fasting blood samples were collected on the second morning after hospitalization. Serum LDL-C and serum TG levels and other blood parameters were detected. Direct measured LDL-Cholesterol (direct LDL-C) was measured by a homogenous direct assay (BECKMAN COULTER Chemistry Analyzer AU5800). Indirect LDL-Cholesterol values were calculated using Friedewald’s equation (Friedewald LDL-C) and Sampson’s equation (Sampson LDL-C). Serum total cholesterol, triglycerides, HDL-Cholesterol were measured via colorimetric assays (BECKMAN COULTER Chemistry Analyzer AU5800). Medical history, demographics, and laboratory test results of patients involved in this study were collected via an electronic medical record system. The data collection protocol was approved by the Institutional Review Committee of Beijing Friendship Hospital Capital Medical University.

### Statistical analysis

This study demonstrated continuous variables as mean ± standard deviation (SD) or median ± interquartile range (IQR). Student’s t-test or Mann–Whitney U-test were employed to compare the difference between groups. Categorical data were illustrated as numbers and sssssssssss. The Pearson chi-square test or Fisher’s exact test was adopted to analyze the difference. The correlations were calculated using Pearson’s correlation test and were shown using a regression curve. Wilcoxon test was applied to estimate the divergence among TG groups. Receiver operator characteristic (ROC) curve analysis was involved to compare the performance of the two equations to evaluate for LDL-C considering the area under the curve (AUC). Bland–Altman plots were used to graphically visualize the consistency and absolute differences between the directly measured LDL-C and the calculated LDL-C, respectively. All statistical tests were performed with IBM SPSS statistics 26 and MedCalc v19.6.4. A two-tailed *P* value < 0.05 was regarded as statistically significant.

## Results

The baseline characteristics are listed in Table 1, the average age was 65 years with a range of measured TG from 24.79 to 2338.47 mg/dL, mean 147.44 mg/dL [0.28–26.41 mmol/l (mean 1.67 mmol/l ± 1.22 SD)]. For all indirect formulas, LDL-C levels depend largely on the TG levels. In order to assess LDL-C levels in the same TG interval, all enrolled patients were divided into six groups, including TG < 100 mg/dL, TG 100–199 mg/dL, TG 200–299 mg/dL, TG 300–399 mg/dL, TG 400–749 mg/dL, TG ≥ 800 mg/dL. Generally, the age of patients decreased along with the TGs increasing, the male percentage increased with increased TGs, BMI levels are relatively higher in patients with high TGs, the number of smokers, hypertension, and diabetes increased with increased TGs. Meanwhile, there is a significant difference of LDL-C value in different TG groups (*P* < 0.001), the LDL-C level gradually increased consistently with the TG levels and reached its peak in TG 300–399 mg/dL group (110.77 ± 31.09 mg/dL), but the LDL-C value decreased with TGs > 400 mg/dL. All the 12,989 ACS patients have evaluated the sets of their lipid profile and the LDL-C levels measured directly is 93.61 ± 29.33, 93.95 ± 35.55 by Friedewald formula, and 97.14 ± 34.27 by Sampson formula (Table 2) Sampson formula exhibits a higher correlation compared with the Friedwald formula (0.943 vs. 0.896).

**Table 1:** Demographic details and clinical characteristics

Variable	Mean ± SD/Median (IQR)							*p* value
	Total population, *n* = 12,989	TG < 100 mg/dl, *n* = 4369	TG 100 ~ 199 mg/dl, *n *= 6402	TG 200 ~ 299 mg/dl, *n* = 1489	TG 300 ~ 399 mg/dl, *n* = 401	TG 400 ~ 799 mg/dl, *n *= 279	TG ≥ 800 mg/dl, *n* = 49	
Age (year)	64.97 ± 10.85	68.00 ± 10.20	64.68 ± 10.63	60.85 ± 10.65	58.68 ± 10.40	57.21 ± 10.56	54.76 ± 10.31	< 0.001
male/female	8430/4559	2895/1474	3992/2410	1006/483	288/113	209/70	40/9	< 0.001
BMI (kg/m^2^)	25.77 ± 3.58	24.69 ± 3.60	26.15 ± 3.47	26.65 ± 3.27	26.84 ± 3.23	27.26 ± 3.62	26.97 ± 3.56	< 0.001
STEMI/NSTEMI/UAP	2018/2010/8961	639/623/3107	1004/980/4418	235/252/1002	76/86/239	55/61/163	9/8/32	< 0.001
smoker	6853 (53%)	2173 (50%)	3335 (52%)	891 (60%)	243 (61%)	179 (64%)	32 (65%)	< 0.001
TG (mg/dl)	147.44 ± 107.99	76.25 ± 15.38	139.35 ± 27.62	235.96 ± 26.50	341.64 ± 28.21	525.04 ± 100.55	1121.12 ± 346.75	< 0.001
TC (mg/dl)	165.28 ± 40.79	148.93 ± 34.80	168.54 ± 38.46	181.51 ± 40.50	194.06 ± 43.55	203.30 ± 51.10	251.02 ± 65.07	< 0.001
LDL-C (mg/dl)	93.61 ± 29.33	81.27 ± 25.31	97.47 ± 28.47	105.25 ± 29.68	110.77 ± 31.09	109.24 ± 32.85	105.46 ± 27.68	< 0.001
HDL-C (mg/dl)	42.06 ± 10.37	45.21 ± 11.33	41.34 ± 9.58	38.36 ± 8.13	37.51 ± 8.01	36.40 ± 9.47	37.43 ± 17.88	< 0.001
nonHDL-C (mg/dl)	123.22 ± 38.63	103.72 ± 31.06	127.21 ± 35.41	143.15 ± 37.04	156.55 ± 40.17	166.90 ± 44.95	213.59 ± 57.06	< 0.001
RC (mg/dl)	29.61 ± 13.48	22.44 ± 8.01	29.74 ± 10.36	37.90 ± 11.53	45.78 ± 14.22	57.67 ± 18.00	108.13 ± 38.33	< 0.001
TG/HDL-C	4.04 ± 20.78	1.80 ± 0.61	3.57 ± 1.28	6.43 ± 1.56	9.56 ± 2.41	15.52 ± 6.44	81.99 ± 329.18	< 0.001
**Medical history**								
Hypertension	9201 (71%)	2997 (69%)	4600 (72%)	1082 (73%)	287 (72%)	201 (72%)	34 (69%)	0.005
Diabetes	4630 (36%)	1395 (32%)	2322 (36%)	603 (40%)	146 (36%)	142 (51%)	22 (45%)	< 0.001
**Blood glucose index**								
RBG at admission (mmol/l)	8.52 ± 3.85	7.98 ± 3.44	8.59 ± 3.86	9.11 ± 4.13	9.41 ± 4.42	10.56 ± 5.27	10.77 ± 4.10	< 0.001
FPG (mmol/l)	6.10 ± 2.41	5.72 ± 1.84	6.25 ± 2.26	6.77 ± 2.52	7.04 ± 2.75	8.14 ± 3.31	8.75 ± 3.32	< 0.001
HbA1c (%)	6.53 ± 1.41	6.28 ± 1.21	6.55 ± 1.41	6.81 ± 1.58	6.92 ± 1.63	7.56 ± 1.92	7.67 ± 1.72	< 0.001
TyG	8.81 ± 0.66	8.21 ± 0.36	8.90 ± 0.37	9.51 ± 0.34	9.92 ± 0.34	10.47 ± 0.42	11.28 ± 0.49	< 0.001
**Discharge diagnose**								
Diabetes	5164 (40%)	1492 (34%)	2583 (40%)	692 (47%)	193 (48%)	175 (63%)	29 (59%)	< 0.001

*BMI* body mass index, *STEMI* ST-elevated myocardial infarction, *NSTEMI* non-ST elevated myocardial infarction, *UAP* unstable angina pectoris, *TC* total cholesterol, *TG* triacylglycerol, *LDL-C* low density lipoprotein cholesterol, *HDL-C* high density lipoprotein cholesterol, *RBG* random blood glucose, *FPG* fast plasma glucose, *HbA1c* hemoglobin A1c, *TyG* triglyceride-glucose index

**Table 2:** Mean values of various methods LDL-C and correlation with direct LDL-C

LDL-C	Formula	Mean ± SD mg/dl (mmol/l)	Mean difference mg/dl (mmol/l)	Correlation (r)	*p* value
Direct LDL-C	Directly measured	93.61 ± 29.33 (2.42 ± 0.76)	NA	1	< 0.001
Friedewald	LDL-C(mg/dl) = TC - HDL-C - TG/5	93.95 ± 35.55 (2.43 ± 0.92)	0.34 (0.01)	0.896	< 0.001
Sampson	LDL-C(mg/dl) = TC/0.948 - HDL-C/0.971 - (TG/8.56 + TG × Non-HDL-C/2140 - TG^2^/16,100) − 9.44	97.14 ± 34.27 (2.51 ± 0.89)	3.53 (0.09)	0.943	< 0.001

*LDL-C* Low-density lipoprotein cholesterol, *TG* triglyceride

**Fig. 2:**
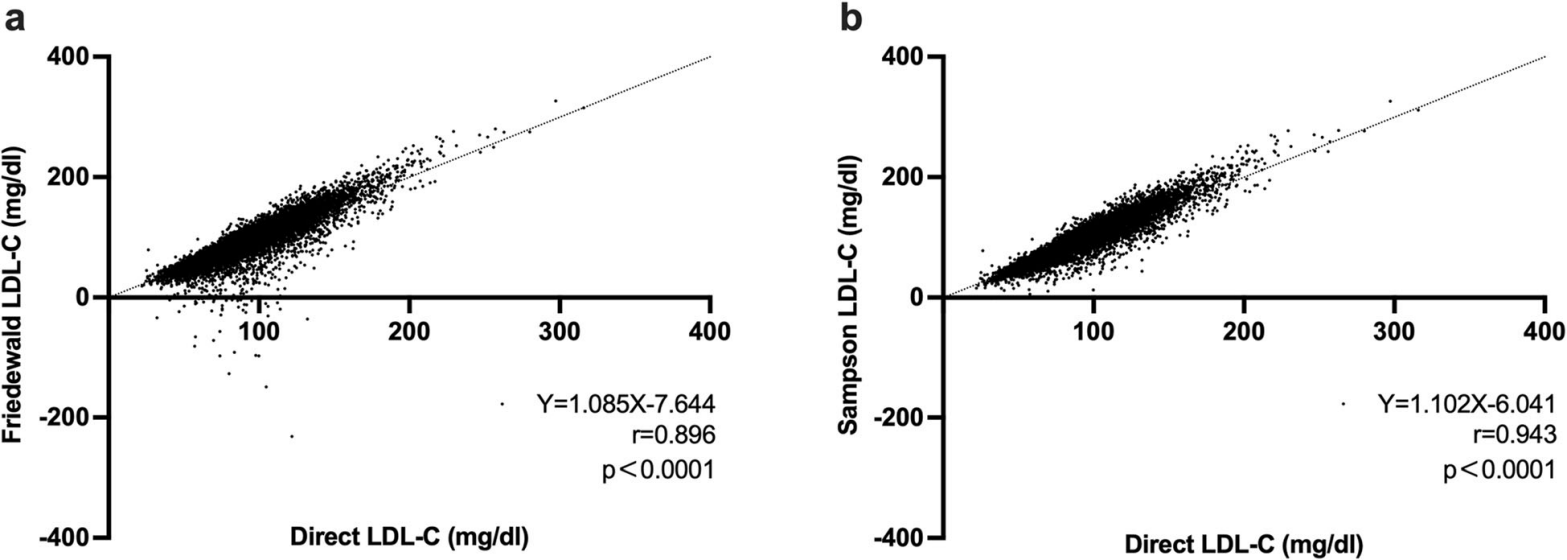
Regression lines between direct LDL-C and LDL-C values estimated with Friedewald’s and Sampson’s formula

**Fig. 3:**
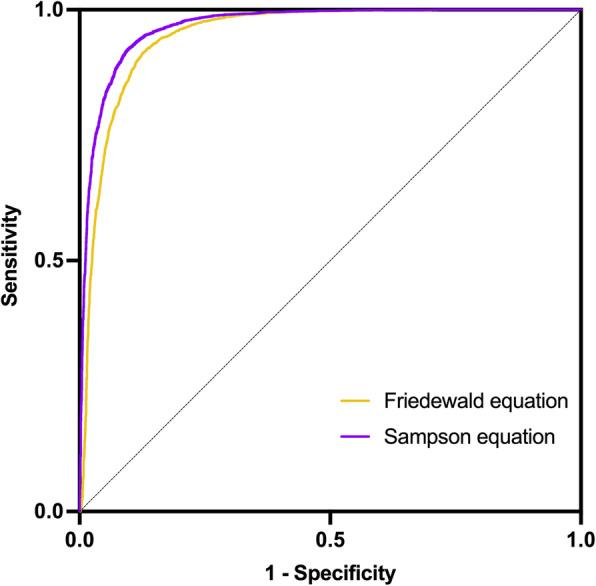
The receiver operating characteristic (ROC) curves of the Friedewald equation and the Sampson equation to measure direct LDL-C levels in patients with ACS. The area under ROC curves (AUCs) of the Sampson equation performed better than Friedewald Eq. (0.968 vs. 0.951; *P* < 0.001). The sensitivity of the Sampson equation was 87.3% and the specificity was 94.1%

Then, a comparison of the correlations between the indirect LDL-C formulas (Sampson and Friedewald) and the direct formula was carried out, and the results indicated that the Sampson formula had a higher correlation (*r* = 0.943, *P* < 0.001) than the Friedewald formula (*r* = 0.896, *P* < 0.001) by Pearson’s analysis. Regression lines between direct LDL-C and calculated LDL-C values are reported in Fig. 2. Meanwhile, the ROC curve was used to compare the efficiency of these two formulas to measure LDL-C levels, the Sampson formula performed better with an AUC of 0.968 than the Friedewald (AUC = 0.951). Although the Sampson equation has lower sensitivity (87.3 vs. 89.4), its specificity is higher than the Friedwald Eq. (94.1 vs. 88.6) Table 3, and the Sampson formula acquired a higher cut-off comparing with Friedwald formula (99.78 vs. 93.32) based on a direct LDL-C median cut-off of 90.87 mg/dL (Fig. 3).

**Table 3:** ROC curve of LDL-C calculated vs. measured LDL-C for the Friedewald, Sampson formula

Formula	Sensitivity (%)	Specificity (%)	cut-off	AUC	(95%confidence internal)
Friedewald	89.4	88.6	93.32	0.951	(95%CI 0.947, 0.954)
Sampson	87.3	94.1	99.78	0.968	(95%CI 0.965, 0.970)

*ROC* the receiver operating characteristic, *AUC* the area under ROC curves

**Fig. 4:**
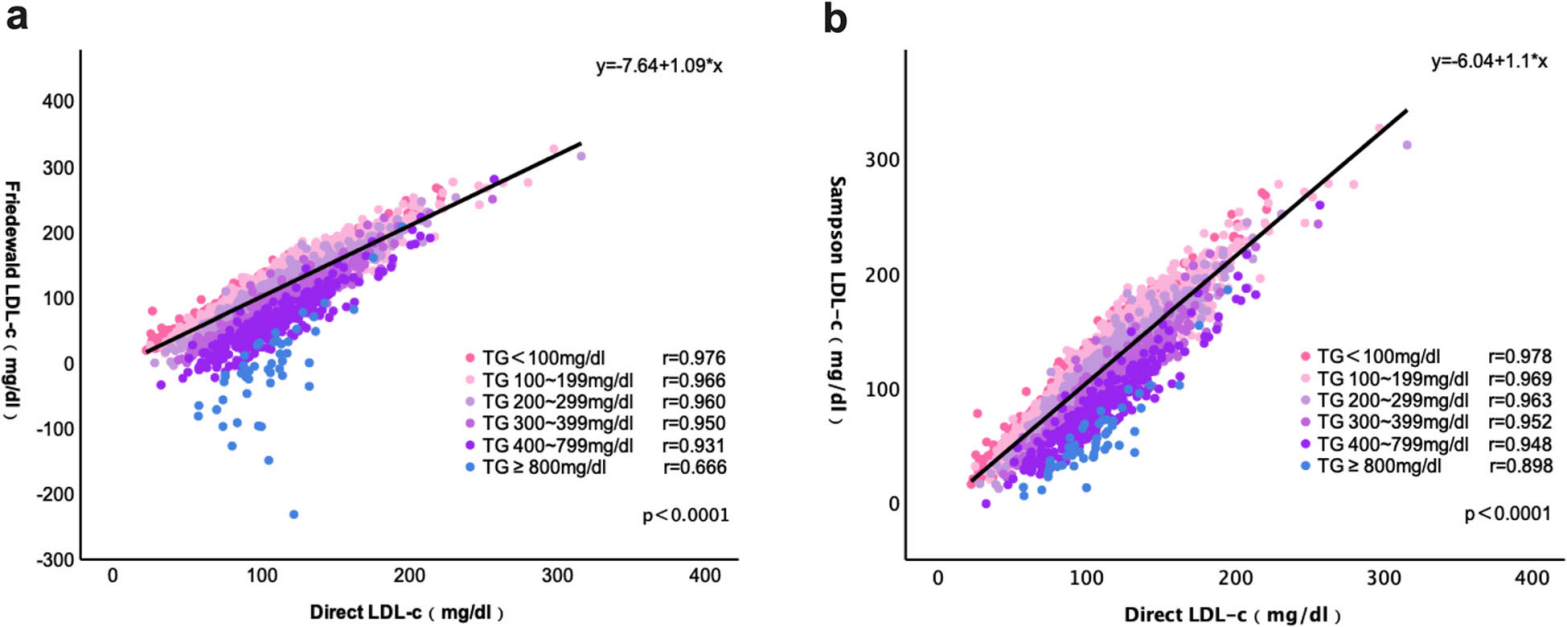
Regression lines between direct LDL-C and LDL-C values estimated with Friedewald’s and Sampson’s formula in different TG groups

**Fig. 5:**
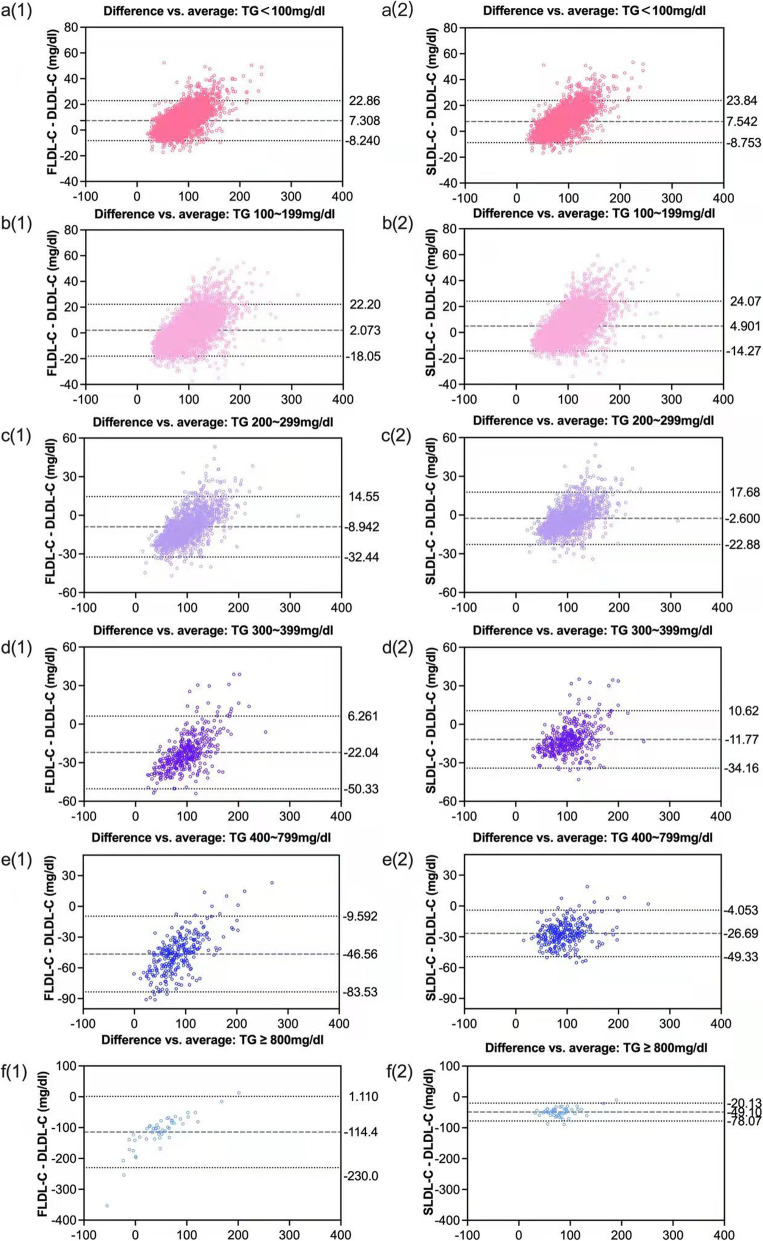
Bland-Altman charts in different TG groups with difference between LDL evaluation methods and their means (Lines represent the average difference between the measurements, the upper and lower control limits of plus and minus 1.96*sigma, respectively, where sigma is the standard deviation of the measurement differences)

Furthermore, the values of LDL-C (mean and SD) were analyzed in all TG level-classified groups (direct assay, Friedewald, and Sampson). For those patients with TG < 200 mg/dL, direct measurement of LDL-C was lower than indirect calculation (Table 4). While with the increased TG levels, the LDL-C values from direct measurement were higher than Friedewald and sssssssss equations. The results of Sampson formulas had a better correlation(r) with direct measurement than Friedewald formulas results in all spectrums of TG, especially in patients with TG > 400 mg/dL. In addition, Sampson exhibits an overwhelming advantage over the Friedewald formula through all spectrums (*P* < 0.001) (Fig. 4), with the increasing levels of TGs, the correlation of Sampson is higher than the Friedewald (0.898 vs. 0.666 in TG ≥ 800 mg/dL, 0.948 vs. 0.931 in TG 400–799 mg/dL, respectively). This is also demonstrated by the Bland-Altman plots, showing that the gap between the two methods increases along with increasing TG levels (Fig. 5).

**Table 4:** Correlation between the calculated LDL-C and directly measured LDL-C in different TG groups

Group	Method	Mean ± SD mg/dl (mmol/l)	Mean difference mg/dl (mmol/l)	Correlation (r)	*p* value
TG < 100 mg/dl, *n* = 4369					
	Direct LDL-C	81.27 ± 25.30 (2.10 ± 0.65)			
	Friedewald LDL-C	88.58 ± 30.46 (2.29 ± 0.79)	7.31 (0.19)	0.976	< 0.001
	Sampson LDL-C	88.81 ± 31.20 (2.30 ± 0.81)	7.54 (0.20)	0.978	< 0.001
TG 100 ~ 199 mg/dl, *n* = 6402					
	Direct LDL-C	97.47 ± 28.47 (2.52 ± 0.74)			
	Friedewald LDL-C	99.54 ± 34.67 (2.57 ± 0.90)	2.07 (0.05)	0.966	< 0.001
	Sampson LDL-C	102.37 ± 34.40 (2.65 ± 0.89)	4.90 (0.13)	0.969	< 0.001
TG 200 ~ 299 mg/dl, *n* = 1489					
	Direct LDL-C	105.25 ± 29.68 (2.72 ± 0.77)			
	Friedewald LDL-C	96.30 ± 37.16 (2.49 ± 0.96)	-8.95 (-0.23)	0.960	< 0.001
	Sampson LDL-C	102.65 ± 35.11 (2.65 ± 0.91)	-2.60 (-0.07)	0.963	< 0.001
TG 300 ~ 399 mg/dl, *n *= 401					
	Direct LDL-C	110.77 ± 31.09 (2.86 ± 0.80)			
	Friedewald LDL-C	88.73 ± 40.19 (2.29 ± 1.04)	-22.04 (-0.57)	0.950	< 0.001
	Sampson LDL-C	99.00 ± 35.90 (2.56 ± 0.93)	-11.77 (-0.30)	0.952	< 0.001
TG 400 ~ 799 mg/dl, *n* = 279					
	Direct LDL-C	109.24 ± 32.85 (2.82 ± 0.85)			
	Friedewald LDL-C	62.68 ± 45.18 (1.62 ± 1.17)	-46.56 (-1.20)	0.931	< 0.001
	Sampson LDL-C	82.55 ± 36.04 (2.13 ± 0.93)	-26.69 (-0.69)	0.948	< 0.001
TG ≥ 800 mg/dl, *n* = 49					
	Direct LDL-C	105.46 ± 27.68 (2.72 ± 0.72)			
	Friedewald LDL-C	-8.96 ± 73.66 (-0.23 ± 1.91)	-114.42 (-2.95)	0.666	< 0.001
	Sampson LDL-C	56.36 ± 33.24 (1.46 ± 0.86)	-49.10 (-1.26)	0.898	< 0.001

*TG* triacylglycerol, *LDL-C* low density lipoprotein cholesterol

**Fig. 6:**
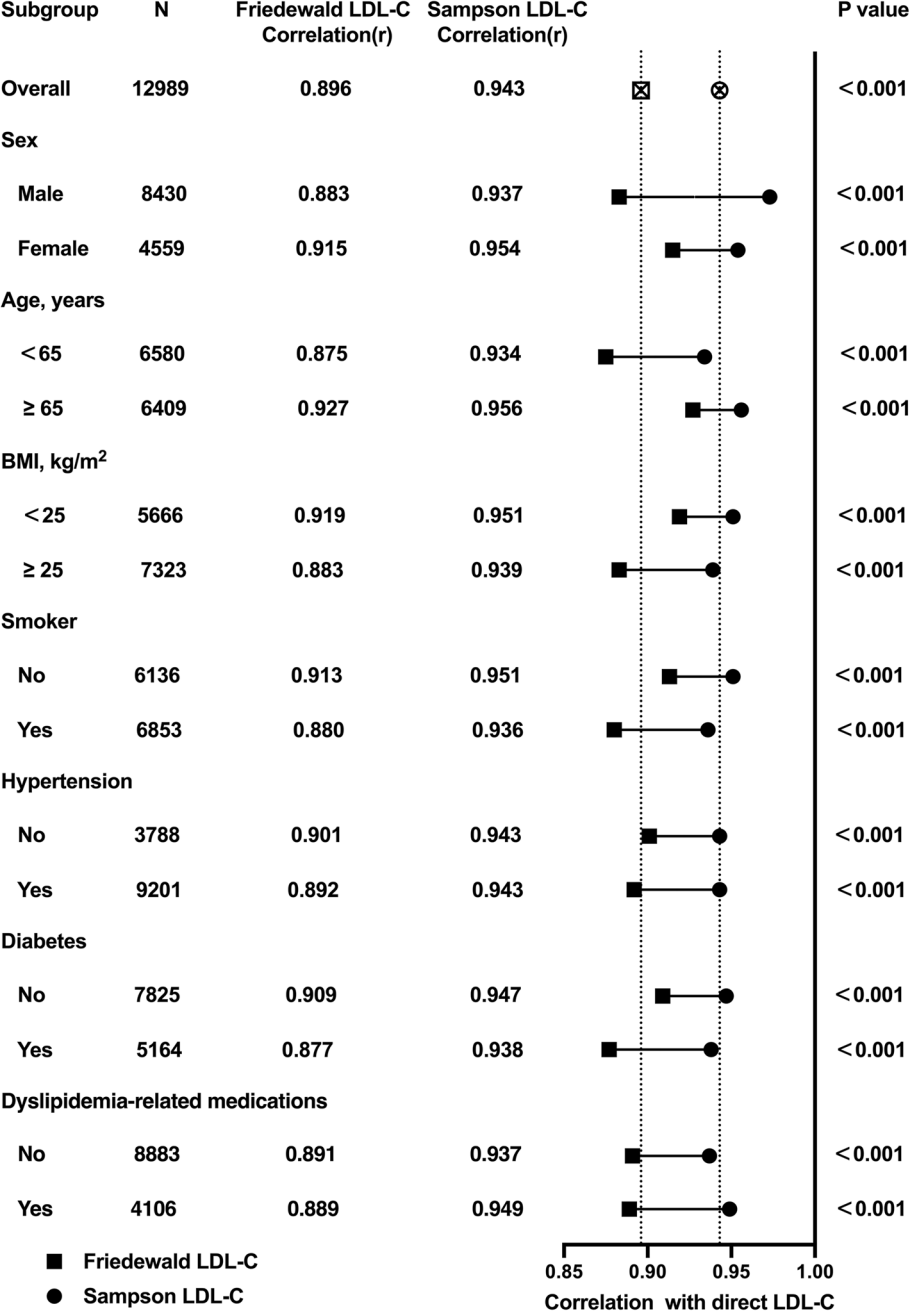
Correlations of the direct LDL-C and LDL-C values estimated with Friedewald’s and Sampson’s equation in all groups under different characteristics or comorbidities

To elucidate the relationship between clinical comorbidities and indirect LDL-C formulas, the following analysis enrolled several clinical factors (including gender, age, BMI, smoker, hypertension, and diabetes) and compared the Friedewald and Sampson formulas (Table 5). The results indicated that the correlation of the Sampson equation is higher in all groups under different characteristics or comorbidities than the Friedewald equation. It shows more advantages in female, age ≥ 65, BMI < 25, non-smoker, and non-diabetes (0.954 vs. 0.937, 0.956 vs. 0.934, 0.951 vs. 0.939, 0.951 vs. 0.936, and 0.947 vs. 0.938, respectively) than those in male, age < 65, BMI ≥ 25, smoker, and diabetes. Whether suffering hypertension or not has the same correlation of Sampson equation (Fig. 6). In addition, for patients with age < 65, BMI ≥ 25, smoker, hypertension, and diabetes, the difference of the correlation between the Friedewald and Sampson equations are larger. Overall, the Sampson formula is better than the Friedewald formula in detecting LDL-C levels under complicated clinical conditions.

**Table 5:** Correlation between the calculated LDL-C and directly measured LDL-C in different groups

	Method	Mean ± SD mg/dl (mmol/l)	Mean difference mg/dl (mmol/l)	Correlation (r)	*p* value
male, *n* = 8430					
	Direct LDL-C	91.97 ± 28.66 (2.38 ± 0.74)			
	Friedewald LDL-C	91.49 ± 34.78 (2.37 ± 0.90)	-0.48 (-0.01)	0.883	< 0.001
	Sampson LDL-C	94.78 ± 33.40 (2.45 ± 0.86)	2.81 (0.07)	0.937	< 0.001
female, *n* = 4559					
	Direct LDL-C	96.64 ± 30.32 (2.50 ± 0.78)			
	Friedewald LDL-C	98.50 ± 36.50 (2.55 ± 0.94)	1.86 (0.05)	0.915	< 0.001
	Sampson LDL-C	101.50 ± 35.42 (2.62 ± 0.92)	3.86 (0.12)	0.954	< 0.001
age ≥ 65, *n* = 6409					
	Direct LDL-C	89.97 ± 27.99 (2.33 ± 0.72)			
	Friedewald LDL-C	92.32 ± 33.21 (2.39 ± 0.86)	2.35 (0.06)	0.927	< 0.001
	Sampson LDL-C	94.70 ± 32.81 (2.45 ± 0.85)	4.73 (0.12)	0.956	< 0.001
age < 65, *n* = 6580					
	Direct LDL-C	97.15 ± 30.17 (2.51 ± 0.78)			
	Friedewald LDL-C	95.54 ± 37.63 (2.47 ± 0.97)	-1.61 (-0.04)	0.875	< 0.001
	Sampson LDL-C	99.51 ± 35.48 (2.57 ± 0.92)	2.36 (0.06)	0.934	< 0.001
BMI ≥ 25, *n* = 7323					
	Direct LDL-C	94.80 ± 29.73 (2.45 ± 0.77)			
	Friedewald LDL-C	93.36 ± 36.48 (2.41 ± 0.94)	-1.44 (-0.04)	0.883	< 0.001
	Sampson LDL-C	97.10 ± 34.65 (2.51 ± 0.90)	2.30 (0.06)	0.939	< 0.001
BMI < 25, *n* = 5666					
	Direct LDL-C	92.06 ± 28.74 (2.38 ± 0.74)			
	Friedewald LDL-C	94.72 ± 34.29 (2.45 ± 0.89)	2.66 (0.07)	0.919	< 0.001
	Sampson LDL-C	97.19 ± 33.78 (2.51 ± 0.87)	5.13 (0.13)	0.951	< 0.001
Smoker, *n* = 6853					
	Direct LDL-C	93.48 ± 29.24 (2.42 ± 0.76)			
	Friedewald LDL-C	92.80 ± 35.48 (2.40 ± 0.92)	-0.68 (-0.02)	0.880	< 0.001
	Sampson LDL-C	96.24 ± 33.95 (2.49 ± 0.88)	2.76 (0.07)	0.936	< 0.001
non-Smoker, *n* = 6136					
	Direct LDL-C	93.75 ± 29.44 (2.42 ± 0.76)			
	Friedewald LDL-C	95.23 ± 35.58 (2.46 ± 0.92)	1.48 (0.04)	0.913	< 0.001
	Sampson LDL-C	98.15 ± 34.60 (2.54 ± 0.89)	4.40 (0.12)	0.951	< 0.001
Hypertension, *n* = 9201					
	Direct LDL-C	92.16 ± 28.91 (2.38 ± 0.75)			
	Friedewald LDL-C	91.91 ± 35.09 (2.38 ± 0.91)	-0.25 (0.00)	0.892	< 0.001
	Sampson LDL-C	95.18 ± 33.77 (2.46 ± 0.87)	3.02 (0.08)	0.943	< 0.001
non-Hypertension, *n* = 3788					
	Direct LDL-C	97.13 ± 30.05 (2.51 ± 0.78)			
	Friedewald LDL-C	98.89 ± 36.17 (2.56 ± 0.94)	1.76 (0.05)	0.901	< 0.001
	Sampson LDL-C	101.90 ± 35.03 (2.64 ± 0.91)	4.77 (0.13)	0.943	< 0.001
Diabetes, *n* = 5164					
	Direct LDL-C	92.32 ± 29.66 (2.39 ± 0.77)			
	Friedewald LDL-C	90.61 ± 36.42 (2.34 ± 0.94)	-1.71 (-0.05)	0.877	< 0.001
	Sampson LDL-C	94.40 ± 34.49 (2.44 ± 0.89)	2.08 (0.05)	0.938	< 0.001
non-Diabetes, *n *= 7825					
	Direct LDL-C	94.45 ± 29.09 (2.44 ± 0.75)			
	Friedewald LDL-C	96.16 ± 34.79 (2.49 ± 0.90)	1.71 (0.05)	0.909	< 0.001
	Sampson LDL-C	98.95 ± 34.01 (2.56 ± 0.88)	4.50 (0.12)	0.947	< 0.001
dyslipidemia-related medications, *n* = 4106					
	Direct LDL-C	84.42 ± 26.77 (2.18 ± 0.69)			
	Friedewald LDL-C	82.91 ± 33.03 (2.14 ± 0.85)	-1.51 (-0.04)	0.889	< 0.001
	Sampson LDL-C	86.14 ± 31.58 (2.23 ± 0.82)	1.72 (0.05)	0.949	< 0.001
No dyslipidemia-related medications, *n* = 8883					
	Direct LDL-C	97.85 ± 29.50 (2.53 ± 0.76)			
	Friedewald LDL-C	99.05 ± 35.52 (2.56 ± 0.92)	1.20 (0.03)	0.891	< 0.001
	Sampson LDL-C	102.22 ± 34.28 (2.64 ± 0.89)	3.47 (0.11)	0.937	< 0.001

*BMI* body mass index, *LDL-C* low density lipoprotein cholesterol

## Discussion

The clinical management of patients with cardiovascular diseases and an accurate estimation of 10-year risk are critically dependent on their level of LDL-C [18]. Maureen et al. reported the new Sampson equation to estimate LDL-C more accurately than traditional methods. This study verified that the Sampson formula is more accurate than the Friedewald formula regardless of patient characteristics and comorbidities, especially in patients with TGs higher than 800 mg/dL.

 As guidelines emphasize the necessity of treating patients with high LDL-C levels to achieve risk stratification goals, it is crucial to accurately evaluate LDL-C levels in patients suffering high cardiovascular risk. Although the test for directly measuring LDL-C has been continuously developed and improved, it remains common practice to calculate LDL-C using indirect formulas. The Friedewald formula is a widely employed indirect equation with the advantages of being rapid, and simple. However, current evidence indicates that the Friedewald equation may underestimate LDL-C levels, especially in patients with TGs > 400 mg/dL[19]. This may be due to the equation: LDL-C = Total cholesterol - HDL-C - TG/5, and a higher TG level may decrease the LDL-C level [10]. Maureen and colleagues developed the new Sampson equation as a more accurate calculation of LDL-C in patients with hypertriglyceridemia but not familial hypertriglyceridemia. It has been reported that the Sampson equation could reduce the possibility of misclassification of patients with hypertriglyceridemia by 35% compared with the Friedewald Eq. 1 [7].

To our knowledge, this is the first study to identify the efficiency of the Sampson equation in Chinese patients. Considering the population characteristics, it’s necessary to identify the application of this equation in Chinese. This study found that the Sampson equation was superior to the Friedewald equation in all TG spectra. One advantage of this study over the work by Maureen et al. is that this study enrolled 49 patients with TG > 800 mg/dL and verified that the Sampson was better than the Friedewald equation in patients with TG > 800 mg/dL thereby extending the application of the Sampson equation. Although the sample was relatively small, it also proved that the Sampson equation is effective in patients with TG > 800 mg/dL. Considering that the LDL-C levels are substandard in Chinese patients, these conclusions may help clinical physicians to develop proper strategies and improve the outcomes of patients with cardiovascular diseases.

This study indicated that although LDL-C measured using the Friedewald equation was closer to the direct level, the correlation was lower than that of the Sampson equation. With the increase in TG levels, the LDL-C values from the Sampson equation were much closer to those in the real world, with a higher correlation. We went through the data and found that the variation in VLDL-C and triglyceride values resulted in this situation, which may underestimate real LDL-C levels in patients with high TGs. In addition, in this clinical study, the Sampson equation has advantages over the Friedewald equation for different clinical characteristics, including age, BMI, sex, hypertension, diabetes, and smoking, which were identified to be closely associated with LDL-C levels.

 This study added relatively powerful evidence of the Sampson equation providing a more accurate estimate of LDL-C values than the Friedewald equation. In addition, compared to other studies [20–22], this study enrolled patients diagnosed with ACS, which has not been reported in other papers. Although this equation is still inadequate for use in clinical practice, further studies are necessary to analyze the efficiency of the equations in different subgroups and proved the superiority of the Sampson equation in various conditions.

## Strength and Limitations

One strength of this study is that the Sampson equation is a novel equation that was first reported in 2020. One of the key conclusions of the work by Maureen et al. is that their equation was more accurate for triglyceride levels up to 800 mg/dL. Relatively, another strength of the work includes: extending indications of the equation to patients (TGs > 800 mg/dL). Furthermore, this study was the first to introduce the Sampson equation to the Chinese population and the results indicated that this new formula was suitable for Chinese patients. This study also has some limitations; one is that all patients enrolled in this clinical retrospective design study were from a single-center, and more studies are necessary to identify the efficiency of the Sampson equation in Chinese patients. Another limitation is that this study only enrolled 49 (0.37%) patients with TG > 800 mg/dL and 328 (2.5%) patients with TG > 400 mg/dL, which may reduce the efficiency of the Sampson equation in patients with hypertriglyceridemia.

## Conclusions

The Sampson equation is a newly developed LDL-C equation for LDL-C measurement. Its efficacy was identified in Chinese patients with hypertriglyceridemia (even TG > 800 mg/dL) in this study, despite patient characteristics and comorbidities. More accurate measurement of the LDL-C levels with the Sampson equation could guide physicians to develop more effective therapeutic strategies.

## Data Availability

The datasets used and/or analyzed during the current study are available from the corresponding author on reasonable request.
